# Supplementary material for: MetaRibo-Seq measures translation in microbiomes
Source: Nat Commun. 2020 Jun 29;11:3268. doi: 10.1038/s41467-020-17081-z (PMC7324362; doi:10.1038/s41467-020-17081-z)
Supplement: Supplementary file 10 — Supplementary Data 7 [file 41467_2020_17081_MOESM10_ESM.zip › File2/Confidence_High_Taxonomy/942_out.krona.html]

Javascript must be enabled to view this page.

members
magnitude
magnitudeUnassigned
count
unassigned
taxon
rank

942\_out

22

22
2
superkingdom

phylum
976
22

22
200643
class

171549
22
order

21
815
family

816
21
1
genus

SRS100907\_contig\_number\_contig-100\_16147.58204

species

SRS047044\_contig\_number\_contig-100\_981.102305
820
1

species

SRS063985\_contig\_number\_13953SRS148159\_contig\_number\_13643
2
1262739


SRS011239\_contig\_number\_19945SRS013382\_contig\_number\_730SRS014534\_contig\_number\_contig-100\_524.14609SRS015065\_contig\_number\_20263SRS015663\_contig\_number\_35612SRS015782\_contig\_number\_contig-100\_1903.256419SRS017375\_contig\_number\_contig-100\_576.577SRS023715\_contig\_number\_19036SRS047433\_contig\_number\_21604SRS051031\_contig\_number\_contig-100\_1016.183838SRS077641\_contig\_number\_contig-100\_2482.2483SRS1041134\_contig\_number\_662SRS1041164\_contig\_number\_4532SRS1055083\_contig\_number\_8862SRS143070\_contig\_number\_15141SRS147271\_contig\_number\_10582SRS150029\_contig\_number\_5773
species
310298
17

family
171550
1

genus
239759
1

626932
1
species

SRS055017\_contig\_number\_31973
